# Patients with rheumatoid arthritis have an altered circulatory aggrecan profile

**DOI:** 10.1186/1471-2474-9-74

**Published:** 2008-05-28

**Authors:** Jean C Rousseau, Eren U Sumer, Gert Hein, Bodil C Sondergaard, Suzi H Madsen, Christian Pedersen, Thomas Neumann, Andreas Mueller, Per Qvist, Pierre Delmas, Morten A Karsdal

**Affiliations:** 1 Inserm Unit 831, University of Lyon, Pavillon F, Hôpital E. Herriot, Lyon, France; 2 Nordic Bioscience, Herlev, Denmark; 3 Division of Rheumatology & Osteology, Department of Internal Medicine III, Friedrich-Schiller University Jena, Germany; 4 Orthopaedic Surgery Unit, Farsoe, Denmark

## Abstract

**Background:**

Rheumatoid arthritis (RA) is a chronic auto-immune disease with extensive articular cartilage destruction. Aggrecan depletion, mediated by aggrecanases is one of the first signs of early cartilage erosion. We investigated, whether measurement of aggrecan and fragments thereof in serum, could be used as biomarkers for joint-disease in RA patients and furthermore characterized the fragments found in the circulation.

**Methods:**

The study consisted of 38 patients, 12 males (62.2 ± 16.0 years) and 26 females (59.8 ± 20.7 years) diagnosed with RA: 41.5 ± 27.5 mm/h erythrocyte sedimentation rate (ESR), 38.4 ± 34.7 mg/ml C-reactive protein (CRP) and 4.8 ± 1.7 disease activity score (DAS) and 108 healthy age-matched controls. Aggrecan levels were measured using two immunoassays, i.e. the ^374^ARGSVI-G2 sandwich ELISA measuring aggrecanase-mediated aggrecan degradation and the G1/G2 sandwich assay, detecting aggrecan molecules containing G1 and/or G2 (total aggrecan) We further characterized serum samples by western blots, by using monoclonal antibodies F-78, binding to G1 and G2, or by BC-3, detecting the aggrecanase-generated N-terminal ^374^ARGSVI neo-epitope.

**Results:**

Total aggrecan levels in RA patients were significantly decreased from 824.8 ± 31 ng/ml in healthy controls to 570.5 ± 30 ng/ml (31% decrease, P < 0.0001), as measured by the G1/G2 ELISA. Western blot analysis with F-78 showed one strong band at 10 kDa, and weaker bands at 25 and 45 kDa in both healthy controls and RA patients. In contrast, staining for aggrecanase-activity revealed only one strong band in RA patients of 45 kDa.

**Conclusion:**

This is the first study, which characterizes different aggrecan fragments in human serum. The data strongly suggests that total aggrecan levels, i.e. aggrecan molecules containing G1 and/or G2 are lower in RA patients, and that RA patients have at least one specific subpopulation of aggrecan fragments, namely aggrecanse generated ^374^ARGSVI fragments. Further clinical studies are needed to investigate the potential of G1/G2 as a structure-related biochemical marker in destructive joint-diseases.

## Background

Rheumatoid arthritis (RA) is a chronic auto-inflammatory disease, which causes functional disability of the joints [1]. The continuous inflammatory processes lead to extensive remodelling and destruction of the joint-architecture [1]. The pathology of the disease is characterized by release of T-cell, macrophage and stromal cell-related cytokines in the synovial fluid. The local signalling as a consequence to these inflammatory molecules results in the expression of an vast array of protease and consequent degradation of extracellular matrix (ECM) of the articular cartilage and the adjacent bone [1,2].

Today, medical intervention of RA relies on identifying drugs targeting joint-pain and swelling caused by the aforementioned inflammatory processes, as well as halting radiological progression and damage of the joints [3]. In this matter, one cytokine, which has been given special focus is tumour necrosis factor alpha (TNFα) [3,4]. The rationale for developing anti-TNFα treatments in RA is based on multiple *in vitro *and *in vivo *studies, showing the excessive stimulatory effect of TNFα on a wide range of biological processes leading to increased inflammation and tissue destruction [3,4].

The predominant constituents of articular cartilage is collagen type II (60–70% of dry weight) and proteoglycans (10% of dry weight) of which aggrecan is the main proteoglycan. Aggreccan is organized into three globular domains, G1, G2 and G3. Loss of the inter globular domain (IGD), which is located between G1 and G2 has the most deleterious effects to proper tissue-function, due to loss of the main glycosaminoglycan (GAG) region [5-8]. The key mediators of cartilage degradation include the MMPs (Matrix Metallo Proteases) and the closely related ADAM-TS (a disintegrin and metalloproteinase with thrombospondin motifs) [10]. The degradation of aggrecan by MMPs and ADAM-TS, results in among other fragments the ^342^FFGVG and ^374^ARGS [10] neo-epitopes, that may be used to monitor cartilage degradation.

An *ex vivo *model for investigating the depletion of the ECM components is the cartilage explant model. Cartilage explants provide a robust system with a high *in vivo-*likeness where the chondrocytes are anchored in the natural ECM [9,10]. Articular cartilage explants subjected to pro-inflammatory cytokines provide a biologically relevant model to study the time-dependent release of ECM degradation-fragments [10-12].

Until now, the gold standard for clinically diagnosing RA patients has been based on self-reported symptoms, assessment of swollen and tender joints, quantification of levels of rheumatoid factor, anti-CCP auto-antibodies against citrullinated filaggrin peptides, as well as imaging techniques, such as X-ray of the joints. Unfortunately, the use of radiological-methods is not without limitations, as significant joint-damage has taken place, by the time, signs of joint-destruction are detected by X-ray [13,14]. Furthermore, radiological imaging of the joints is a cumbersome procedure, where long follow-up periods are necessary in order to assess noticeable changes of the articular cartilage [15]. Markers like C-reactive protein (CRP), matrix metalloproteinase-3 (MMP-3) and YKL-40 have also been used for assessing the extent of synovitis in RA patients [16-19]. But CRP and MMP-3 are not specific to the synovial tissue [20], and though serum YKL-40 levels are elevated in RA individuals, this marker is highly correlated to CRP levels, and therefore may not be of further value to reflect joint-destruction [21]. Consequently, more dynamic sensitive non-invasive markers are desired, which may aid in providing information of the structural damage of the joints and the efficacy of structure-modifying drugs.

In the current study, we have used corresponding immunoassays to investigate, if monitoring of aggrecan, or its fragments in the circulation, can be used as valid biomarkers for assessing joint-damage in RA patients, and moreover, characterized the fragments found in human serum.

## Methods

### Study participants

The clinical data were evaluated by selecting 38 patients, 12 males (62.2 ± 16.0 years), (83.0 ± 13.9 kg), (173.0 ± 6.4 cm) and 26 females (59.8 ± 20.7 years), (164.2 ± 5.8 cm) (64.8 ± 10.9 kg) (mean ± SD) with RA. The eryhthrocyte sedimentation rate (ESR), C-reactive protein (CRP) and disease activity score (DAS)(37) were 41.5 ± 27.5 mm/h, 38.4 ± 34.7 mg/l and 4.8 ± 1.7 respectively. X-ray pictures were evaluated according to the Steinbrocker method [22] and scored 1–4, which was 2 ± 0.9. Either, the patients were untreated, or underwent drug-treatment with different dosages of 1) disease modifying anti-rheumatic drugs (DMARDs): Prednisolone, Sulfasalazin, Methotrexate, Leflunomid, Azathioprin either alone, or in combination (39.5% of the patients were on co-medications), or 2) with non-steroidal anti-rheumatic drugs: Rofecoxib, Diclofenac, Valdecoxib, Acemetacin, Piroxicam, Celecoxib, Ibuprofen alone. The majority of the patients were positive for rheumatoid factors and typical rheumatoid nodes. A set of 108 healthy age-matched males and females were used as controls for the RA patients (60.6 ± 13.9 years). The study was approved by the Ethical Committee of Medical Faculty at Friedrich-Schiller University (Jena, Germany-reference number 1019-01/03) and written patient consent was received for the study. Blood was collected from fasting individuals in the morning, and allowed to clot at room temperature for at least 10 minutes. Then the collected blood was centrifuged for 10 minutes at 1000 g and serum was harvested, and stored at -70°C, before analysis. For Western Blot analysis, 2 groups of patients were randomly selected: 15 plasma samples from RA patients (61 ± 3.6 years, DAS >5) and 22 healthy age-matched (62 ± 2.7 years) controls.

### Reagents

ADAMTS-4 (Cat. Number CC1028) 5 μg/25 μl and polyclonal rabbit antimouse antibody (Cat. Number AQ160) 1 mg/ml were from Chemicon, USA. Tumour Necrosis Factor alpha (TNFα) 10 μg/ml (Cat. Number 210-TA) was bought from R & D systems. Maxisorp-plates were purchased from Nunc. (Cat. Number 439454), while streptavidin-coated plates were bought from Roche (Cat. Number 1207733). Purified bovine aggrecan 1 mg (Cat. Number A-1960), Oncostatin M 10 μg (OSM) (Cat. Number O 9635-10UG), Peroxidase-conjugated rabbit anti-mouse secondary antibody (Fc specific) (A-2554) were all purchased from Sigma Aldrich, DK, and Liquid II was purchased from Roche. The ELISA assay quantifying the levels of auto-antibodies against citrullinated filaggrin peptides, Diastat Anti-CCP (Cat. Number UK-FCCP200), was purchased from Axis-Shield Diagnostics, UK. Nitrocellulose membrane (Cat. Number FB 0303-1) was bought from Whatman Schleicher and Schuell. Normal freeze-dried mouse serum (Cat. Number NS03L) was bought from Calbiochem. Cell supernatants of the monoclonal antibody BC-3 (Cat. Number ab 3773) against the aggrecanase-generated neo-epitope ^374^ARGSVI was purchased from Abcam. The culture medium for the human articular cartilage explants were Dulbecco's Modified Eagle Medium (D-MEM) and Ham F12 from Life Technologies, US. Penicillin and streptomycin 10000 μg/ml (Cat. number DE 17-602E) was from Invitrogen, DK. The ECL detection system for western blot (Cat. Number RPN 2109) was from Amersham.

### Human articular cartilage

Human articular cartilage was obtained from the orthopaedic surgery unit during knee arthroplasty of both females and males with late-stage OA or RA (Farsoe Nordjylland, Denmark). The articular cartilage was isolated without the adhering subchondral bone. The study was conducted according to the ethical committee approval number VN-2060031. RA and OA knee cartilage in response to the cytokines displayed smilar inductions patterns.

Pieces of cartilage (20–25 mg) were placed in 96 well plates in 4-replicates and incubated for 21 days at 37°C with 5% CO_2 _in serum-free D-MEM medium. Explants were incubated either non-stimulated, or with 10 ng/ml oncostatin M (OSM) in combination with 20 ng/ml tumour necrosis factor alpha (TNFα) [10,23-25] to stimulate cartilage degradation. As negative control, cartilage was placed in cryo-tubes, frozen in liquid N_2_, and thawed at 37°C in water-bath for three repeated freeze-thaw cycles. The explant culture medium was replaced every 3^rd ^day for 21 days. The conditioned medium was stored at -20°C until analysis.

### Detection of aggrecan neo-epitopes

Aggrecanase -mediated aggrecan degradation was quantified using the ^374^ARGSVI-G2 ELISA [26]. Briefly, it is a sandwich assay using monoclonal antibody BC-3, recognizing the aggrecanase generated neo-epitope ^374^ARGSVI, as catching antibody, and POD-labelled monoclonal antibody F-78 binding to the G2 domain as detector antibody [12]. Briefly, microtitre plates are coated overnight with anti-mouse immunoglobulins (rabbit) diluted in Na_2_CO_3 _buffer, washed, incubated with monoclonal antibody BC-3 diluted PBS-BTB (1 hr, 20°C), washed again and then incubated with sample diluted in PBS-BTB (1 hr, 20°C). Purified bovine aggrecan treated with ADAM-TS4 was used as calibrators. After washing, POD-labelled F78 diluted in PBS-BTB was added to each well (1 hr, 20°C), washed and then incubated with TMB. After 15 minutes the reaction is stopped with 0.18 M H_2_SO_4_. The intra and interassay variation of the assay was 9.6% and 11.2%, respectively.

### Detection of aggrecan turnover

The level of released total aggrecan molecules were quantified using the G1/G2 assay. This assay employs F-78 both as the catching and detecting antibody, binding to a repetitive epitope exposed at least twice on G1 and G2, and detects intact G1-G2, or free catabolized G1 or G2 domains [12]. Briefly, microtitre plates precoated with streptavidin are incubated with biotinylated F78 diluted PBS-BTB (1 hr, 20°C), washed again and then incubated with sample diluted in PBS-BTB (1 hr, 20°C). Purified bovine aggrecan was used as calibrators. After washing, POD-labelled F78 diluted in PBS-BTB was added to each well (1 hr, 20°C), washed and then incubated with TMB. After 15 minutes the reaction is stopped with 0.18 M H_2_SO_4_. Testing human serum samples, the G1/G2 ELISA was modified by addition of a HAMA blocking agent, i.e 10% Liquid II, to the buffer used for dilution of sample and detecting antibody. The intra and interassay of the assay was 7.1% and 8.9%, respectively [12]. Dilution recovery was 110.4% (103.9–116.9%) (mean (range)), and spiking recovery was 98.9% (88.9–110.3%).

### Detection of anti-CCP

Anti-CCP-ELISA was used according to the procedure described by the manufacturer (Axis-Shield Diagnostics, UK). Precision data of intra and interassay of the assay is between 4.7–7.2% [27].

### Western blotting

A pool of 15 plasma samples from RA patients, or 22 samples from healthy controls were diluted 10 times with milli-Q water and boiled for 5 minutes and 30 microliter was run in 4–20% pre-casted gels (Bio-Rad) under reducing conditions (migration buffer: Tris 25 mM, Glycine 200 mM, Sodium Dodecyl Sulfate (SDS) 3.5 mM, pH = 8.3). After transferring the proteins to a polyvinyldifluoride (PVDF) membrane overnight at 50 V in a 10 mM CAPS buffer with 5% methanol, the membrane was blocked with 5% non-fat milk in PBS buffer (1.5 mM KH_2_PO_4_, 8 mM Na_2_HPO_4_2H_2_O, 2.7 mM KCl, 150 mM NaCl with 0.05% Tween-20) with shaking for 1 hour at room temperature (RT). After washing, the following antibodies were applied in appropriate dilutions in PBS buffer with 2.5% bovine serum albumin and 0.05% Tween-20 for 4 hours at RT, or overnight (ON) at 4°C: BC-3 cell-supernatants diluted 1:100, or F-78-POD at a final concentration of 2 μg/ml respectively. For testing the specificity of the obtained bands, inhibition tests were performed with the appropriate antigens. For the antigen-blocking tests, the used concentrations were: intact bovine aggrecan at 333 μg/ml and ^374^ARGSVI-peptide at 200 μg/ml. After washing, the membranes were incubated for 1 hour at RT with a peroxidase (POD)-labeled rabbit anti-mouse antibody for BC-3 1:30000, or directly detected for the POD-coupled F-78. The results were visualized using an ECL system.

### Statistics

Results are shown as mean + standard error of mean (SEM). Differences between mean values were compared by the non-parametric two-tailed student's t-test using the GraphPad Prism software. Differences were considered statistical significant, if P < 0.05.

## Results

### Increased release of aggrecan and its fragments after catabolic stimulation of human cartilage explants with pro-inflammatory cytokines

First, the local response of human chondrocytes subjected to catabolic stimuli by exposure to pro-inflammatory cytokines was investigated. For this purpose, OSM was used in combination with TNFα, and the level of aggrecan and its fragments released into the conditioned medium was evaluated by corresponding immuno-assays.

**Figure 1:**
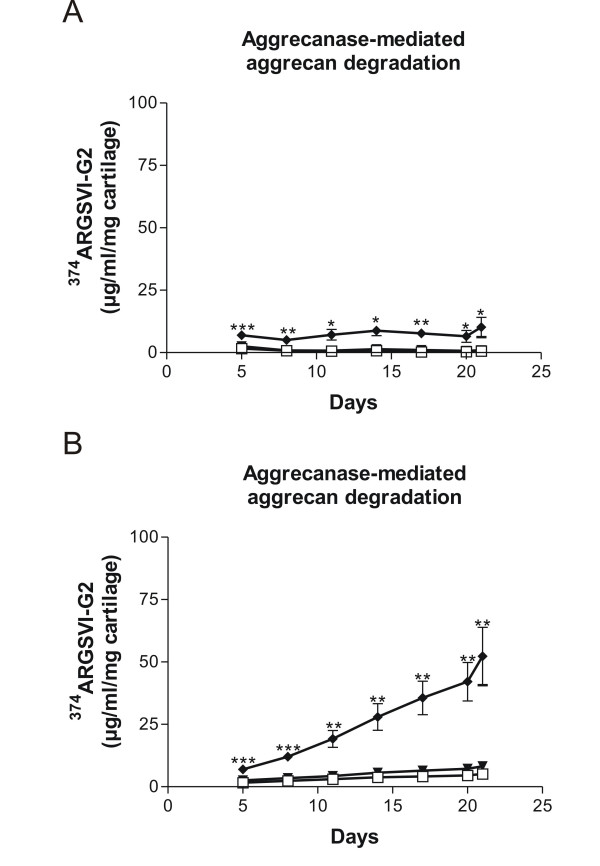
**Measurement of aggrecanase-generated ^374^ARGSVI-G2 fragments in human OA patient**. Human cartilage explants were cultured for 21 days either non-stimulated (-▼-), or treated with pro-inflammatory cytokines 10 ng/ml oncostatin M (OSM) in combination with 20 ng/ml tumour necrosis factor alpha (TNFα) (-◆-). The conditioned medium from four independent wells was measured for the presence of aggrecanase-generated ^374^ARGSVI-G2 fragments at each collected time-point **(a)**, or **(b) **accumulated throughout the study-period. As negative control, explants were frozen and thawed four times in liquid nitrogen (--). The asterisks indicate significant differences (P < 0.05). For the statistical analysis, two-tailed non-parametric t tests were used.

**Figure 2:**
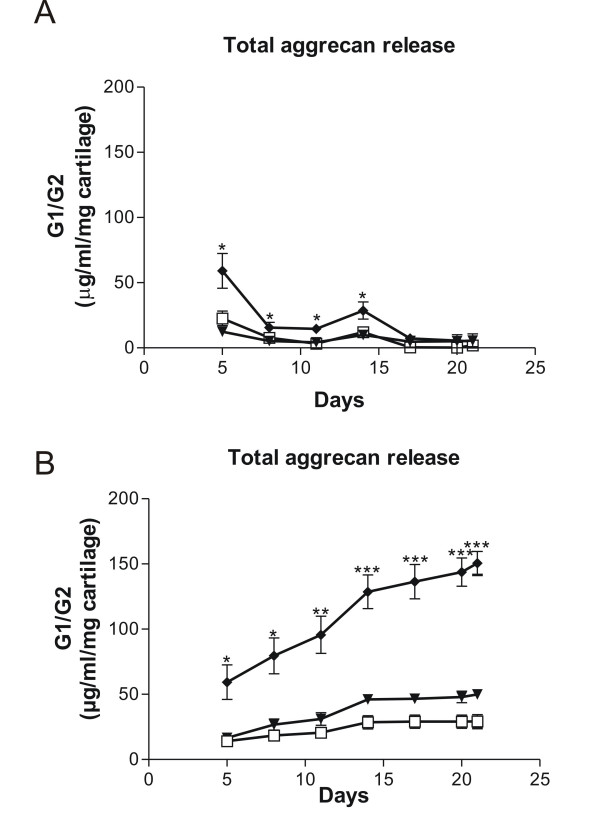
**Measurement of total aggrecan turnover, G1/G2, in human OA patient**. Human cartilage explants were cultured for 21 days either non-stimulated (-▼-), or treated with pro-inflammatory cytokines 10 ng/ml oncostatin M (OSM) in combination with 20 ng/ml tumour necrosis factor alpha (TNFα) (-◆-). The conditioned medium from four independent wells was measured for the presence of G1/G2 molecules at each collected time-point **(a)**, or **(b) **accumulated throughout the study-period. As negative control, explants were frozen and thawed four times in liquid nitrogen (--). The asterisks indicate significant differences (P < 0.05). For the statistical analysis, two-tailed non-parametric t tests were used.

While release of aggrecanase-generated ^374^ARGSVI-G2 fragments were elevated at all time-points in the study-period in response to catabolic stimulation (Figure 1a), the release of G1/G2 could not be detected at late stages in the supernatant (Figure 2a).

The accumulated release of the markers at the end of the study-period resulted in a release of ^374^ARGSVI-G2 and G1/G2, elevated approximately 915% (from 5.15 to 52.27 μg/ml/mg cartilage) and 214% (from 47.83 to 150.19 μg/ml/mg cartilage) respectively compared to vehicle (Figure 1b, 2b).

### Patients with Rheumatoid Arthritis have an altered aggrecan profile compared to healthy controls

To further investigate the aggrecan degradation profile *in vivo *under pathologically relevant conditions, we investigated the systemic levels of total G1/G2 aggrecan molecules in patients diagnosed with RA in order to test the clinical applicability of the assay. For this purpose, a cohort, consisting of 38 patients with RA and 108 healthy age-matched controls was used. Similarly, G1/G2 was compared to the well-recognised RA marker anti-CCP, which is specific for auto-antibodies against citrullinated filaggrin peptides, however not related directly to articular cartilage damage.

**Figure 3:**
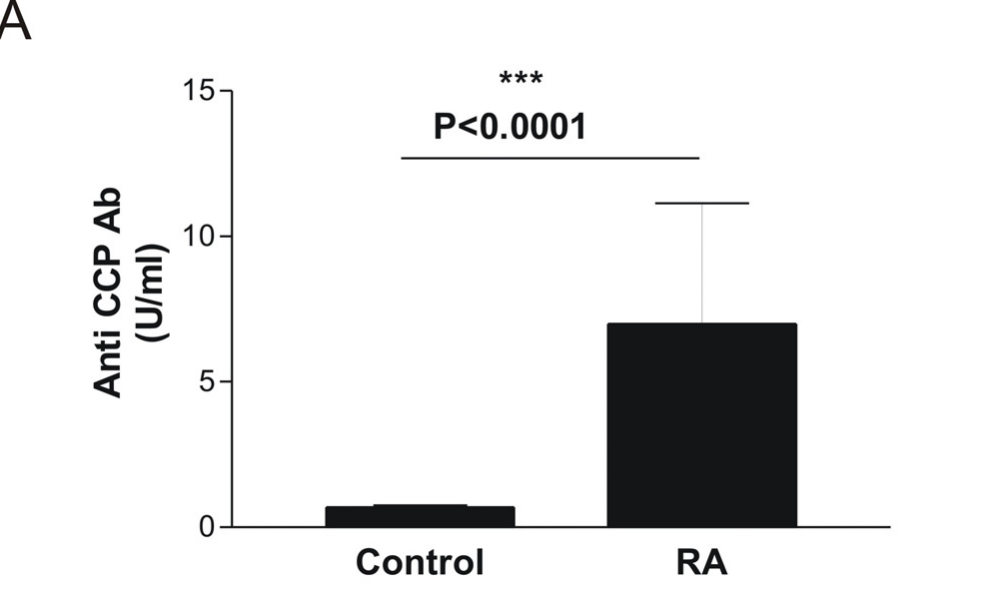
**Quantification of anti-CCP in RA patients**. Serum samples from healthy individuals (N = 104) and patients with Rheumatoid arthritis (RA) (N = 38) were analyzed for their anti-CCP level. The activities are log-transformed data and the values are mean + SEM. The asterisks indicate significant differences (P < 0.05). For the statistical analysis, two-tailed non-parametric t-tests were used.

**Figure 4:**
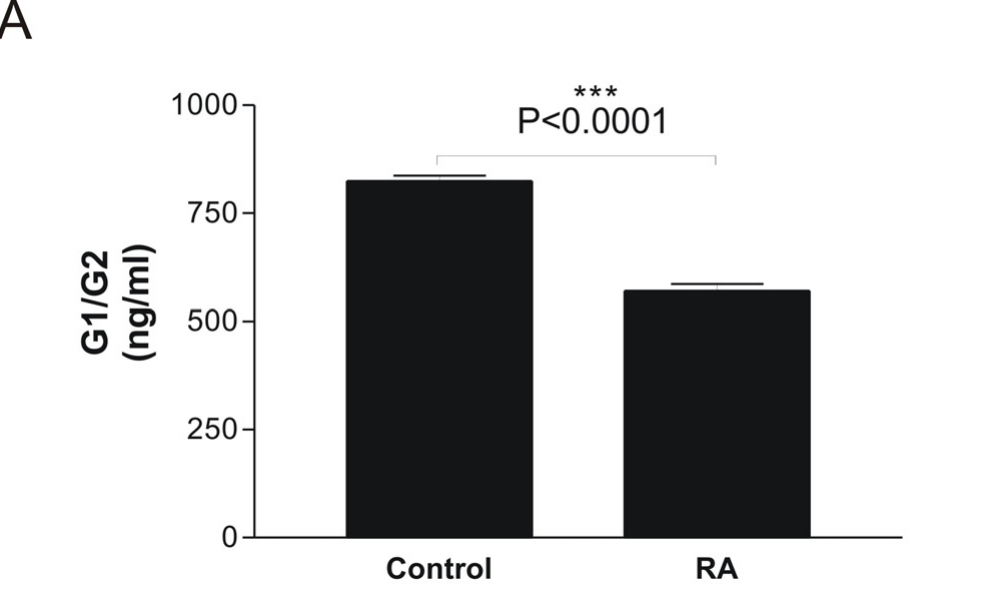
**Quantification of total aggrecan turnover, G1/G2, in RA patients**. Serum samples from healthy individuals (N = 108) and patients with Rheumatoid arthritis (RA) (N = 38) were analyzed in the G1/G2 assay. The concentrations are log-transformed data and the values are mean + SEM. The asterisks indicate significant differences (P < 0.05). For the statistical analysis, two-tailed non-parametric t-tests were used.

The levels of anti-CCP were increased in RA patients (7.00 ± 4.14 U/ml (mean ± SEM) compared to controls (0.69 ± 0.06 U/ml U/ml) (P < 0.0001) (Figure 3), **i.e. **approximately 10 times elevated. On the other hand, the aggrecan turnover was significantly suppressed from 824.8 ng/ml in healthy controls to 570.5 ng/ml, corresponding to a decrease of 31% (P < 0.0001) (Figure 4). In this population of patients with RA, G1/G2 was not correlated to neither ESR, CRP, nor disease activity score.

### Detection of aggrecan fragments in human serum by western blot analysis

The different analytes found in the circulation of aggrecan were characterized by western blotting, where staining was either with F-78, binding to G1 and/or G2 containing molecules, or BC-3, detecting the aggrecanase-generated ^374^ARGSVI N-terminal flanking fragments.

**Figure 5:**
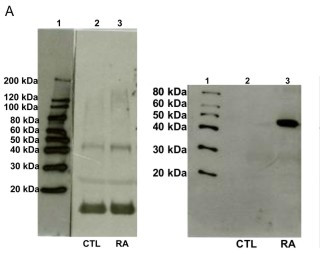
**Characterization of aggrecan fragments in RA patients by western blot analysis**. Western blot analysis was done using 15 independent human plasma samples pooled from patients with Rheumatoid arthritis (RA) (lane 3) and the membrane was stained with **(a) **the monoclonal antibody F-78 raised against intact bovine aggrecan, binding to G1 and G2, or with **(b) **BC-3 raised against the aggrecanase-generated ^374^ARGSVI sequence. As control, plasma samples from 22 healthy individuals were used (lane 2). A standard molecular weight marker was also run to determine the size of the detected fragments (lane 1).

Western blot analysis showed one strong band detected at 10 kDa, and weaker bands at 25 and 45 kDa in both healthy controls and RA patients with F-78 staining (Figure 5a). On the other hand, only one strong aggrecanase-generated ^374^ARGSVI band was present in RA patients of 45 kDa when staining was done with BC-3 (Figure 5b). The binding of both antibodies to the immobilized aggrecan fragments on the membrane was completely inhibited by incubation with the homologous peptides (Data not shown).

## Discussion

Biochemical markers offer the advantage to monitor joint-destruction more dynamically than traditional radiographic techniques, and may therefore allow medical intervention prior to extensive damage of the joints has taken place. Presently, no structure-related markers of articular cartilage proteoglycans have been detected in serum. The primary aim of the current study was to investigate the aggrecan turnover and profilein RA patients receiving treatment. In the present study, we clearly demonstrated that RA patients have significant decrease in circulating levels of aggrecan core-protein containing the G1 and/or G2 domain, and that RA patients have an specific subpopulation aggrecanase degraded aggrecan fragments.

In RA patients, elevated anti-CCP levels were detected, which was expected due to increased inflammation in arthritic individuals (Figure 3). In contrast, the aggrecan turnover was significantly decreased in RA patients compared to controls (Figure 4). Although this effect biologically may be interpreted as extensive loss of articular cartilage, the potential effect drug-treatments cannot be ruled out. The current data needs to be validated in longitudinal studies, to investigate the time dependent release of these aggrecan molecules. In alignment, the clinical potential of the G1/G2 assay to predict structure modifying effects of novel treatments would be of high interest. At present, the sensitivity of the ^374^ARGSVI -G2 ELISA does not allow reliable measurements in human serum to be performed.

When investigating the characteristics of analytes in human serum by western blot analysis, one specific aggrecanase-generated ^374^ARGSVI band of approximately 45 kDa was observed only present in RA serum (Figure 5b). This is in agreement with the general idea that there is an elevated inflammation in the joints of RA individuals due to recruitment of various inflammatory cells that secrete excessive levels of cytokines and growth factors [28,29]. This leads to the expression of a range of proteases of which the ADAM-TS have been show to be the main mediators of aggrecan destruction, that results in the generation and release of the specific fragment of aggercan, ^374^ARGSVI [10]. The band could potentially represent an aggrecan fragment with the ^374^ARGSVI neo-epitope at the N-terminal further comprising the G2 domain at the C-terminal. This would be in alignment with the observation that ^374^ARGSVI-G2 fragments were detected in catabolically stimulated *ex vivo *explant cultures (Figure 1a,b). This fragments may not be connected with the CS1 and CS2 glycosaminoglycan regions, as in this case, they would be expected to give rise to high molecular-weight bands. This is in alignment with that extensive protease activities processes aggercan to smaller fragments.

Though, aggrecan fragments found in the circulation had never been characterized prior to this study, different investigators have analyzed the characteristics of molecules found in OA synovial fluid [30-32]. The N-terminally flanked ^374^ARGSVI neo-epitope was detected in a heterogenous population of fragments in all the above-mentioned studies. The molecular weights detected respectively in these studies were in the range of 90–150 [30], 250 kDa [31] and 129–311 kDa [32]. Struglics and investigators observed that ^374^ARGSVI was linked to the CS-1, but our detection of a band of 45 kDa further suggests a cleavage in the C-terminal part of ^374^ARGSVI-generated fragments in the synovium before reaching the circulation, which could likely be at the CFRG^656^-^657^ISAV MMP-site [32]. However, identical bands of 10, 25 and 45 kDa were observed in both healthy controls, as well as RA patients after staining with F-78 (Figure 5a), different from the G1/G2 ELISA results (Figure 4), indicating that these fragments are a part of normal aggrecan turnover, and therefore, do not reflect a pathological turnover-process. Future efforts of affinity-column-purification of these fragments with F-78 and BC-3 from RA serum, followed by gel-electrophoresis, western blotting and sequencing of the bands may aid in understanding of the molecular mechanisms of normal and pathological aggrecan turnover in joint-debilitating diseases.

To further characterize the aggrecan turnover profile, we used explants from human articular cartilage, and investigated the release of G1/G2, or aggrecanase generated fragment ^374^ARGSVI-G2 aggrecan molecules locally after catabolic treatment. We observed release of ^374^ARGSVI-G2 fragments starting from the initial phases and throughout the whole study-period (Figure 1a), generally demonstrating aggrecanase-activity only at initial time-points [33-36]. The observed differences may be caused by the fact that human articular cartilage from a late-stage OA individual was used in this study, presumably with a high background protease-activity in the matrix, due to the advanced disease stage.

The lack of elevated levels of G1/G2 analytes observed after catabolic treatment of explants at late stages compared to vehicle (Figure 2a) was somehow reflected in the circulation, as the concentrations in RA patients was significantly decreased compared to controls (Figure 4). Collectively, the decrease of aggrecan levels in the RA patients compared to healthy individuals might be a result of the disease being at a progressed stage, where much of the cartilage had already been lost.

There are important limitations associated with current study. Firstly the relative small sample size analysed, and importantly the lack of longitudinal analysis. The current data needs to be validated in longitudinal studies, to investigate the time dependent release of these aggrecan molecules and aggrecan profiles. In addition, preferably in clinical trials, there are a need to investigate the clinical potential of the G1/G2 assay to predict structure changes, and whether the assay might be used as a prognostic marker for progression in both the absence and presence of treatment.

## Conclusion

For the first time, we demonstrate that the aggrecan turnover is significantly decreased in serum of RA patients. Future clinical intervention studies with chondro-protective agents are needed to evaluate its potential as a structure-related marker in destructive joint-diseases.

## Abbreviations

CRP: C-reactive protein, CS1 and CS2: Chondroitin sulphate region 1 and 2, DAS: Disease activity score, DMARDs: Disease modifying anti-rheumatic drugs, ECM: Extracellular matrix, ESR: Eryhthrocyte sedimentation rate, GM-CSF: Granulocyte-macrophage colony-stimulating factor, IGD: Interglobular domain, IL: Interleukin, MMP: Matrix metalloproteinase, OA: Osteoarthritis, ON: Overnight, OSM/TNFα: Oncostatin M and Tumour Necrosis Factor Alpha, POD: Peroxidase, PVDF: Polyvinyldifluoride, RT: Room temperature, RA: Rheumatoid Arthritis, RANK: Receptor Activator of NF-kB, SDS: Sodium dodecyl sulphate, VCAM: Vascular cell adhesion molecule, VEGF: Vascular Endothelial Growth Factor.

## Competing interests

The authors declare that they have no competing interests.

## Authors' contributions

JCR designed and performed the western blot analysis in the study, and participated in drafting of the manuscript. EUS measured anti-CCP and G1/G2 in human serum and quantified ^374^ARGSVI-G2 and G1/G2 levels in the conditioned medium of human OA explants and drafted the manuscript. BCS and SHM designed and performed the articular cartilage explant cultures, and participated in the measurement of biochemical markers of cartilage turnover. GH, TN and AM collected and provided with the RA human serum samples and controls and additional demographic information regarding the individuals. CP performed knee replacement surgery, made ethical committee applications and collected articular cartilage for experimental settings. PQ took part in analysis of data and drafting the manuscript. PD took part in designing of experiments and analyzing data. MAK took part in drafting the manuscript, made the final version of the manuscript, and designed experimental set-up and analysis of data. All the authors have read and approved the final manuscript.

## Pre-publication history

The pre-publication history for this paper can be accessed here:


